# Optimizing Dynamical Network Structure for Pinning Control

**DOI:** 10.1038/srep24252

**Published:** 2016-04-12

**Authors:** Yasin Orouskhani, Mahdi Jalili, Xinghuo Yu

**Affiliations:** 1 Department of Computer Engineering, Sharif University of Technology, Tehran, Iran; 2 Department of Electrical and Computer Engineering, School of Engineering, RMIT University, Melbourne, Australia

## Abstract

Controlling dynamics of a network from any initial state to a final desired state has many applications in different disciplines from engineering to biology and social sciences. In this work, we optimize the network structure for pinning control. The problem is formulated as four optimization tasks: *i*) optimizing the locations of driver nodes, *ii*) optimizing the feedback gains, *iii*) optimizing simultaneously the locations of driver nodes and feedback gains, and *iv*) optimizing the connection weights. A newly developed population-based optimization technique (cat swarm optimization) is used as the optimization method. In order to verify the methods, we use both real-world networks, and model scale-free and small-world networks. Extensive simulation results show that the optimal placement of driver nodes significantly outperforms heuristic methods including placing drivers based on various centrality measures (degree, betweenness, closeness and clustering coefficient). The pinning controllability is further improved by optimizing the feedback gains. We also show that one can significantly improve the controllability by optimizing the connection weights.

Networked structures are found everywhere in our daily life; there exist a network system where a kind of information exchange is happening^1^,^2^. Networks are comprised of two or more nodes that are connected through directed/undirected and weighted/unweighted edges. Examples of everyday life complex network systems include the World Wide Web, the Internet, power grids, transportation and water distribution networks. Complex networks have different definitions, but in the most valid definition we call a network as complex when it has non-trivial structure^3^. A series of research works in network science are based on considering specific dynamics on the nodes and study collective dynamics arising from complex interaction networks^4^,^5^,^6^. It has been shown that if dynamical systems interact over a network and some conditions are met, a collective behavior (synchronization or consensus) emergences.

Controlling dynamics of a network from any initial state to a final desired state has many applications in different fields, which has been heavily studied in network science community^7^,^8^,^9^,^10^,^11^. For example, modern power grids with many generators and consumers are required to be controlled to have desirable performance. The ease by which the whole network can be pinned to a reference state is denoted as its pinning controllability^12^,^13^,^14^. This is often performed by choosing some of the nodes as drivers to which the control signal is fed. The master stability function formalism, developed to study linear stability of the synchronization manifold in coupled identical dynamical systems^15^, is a proper tool to study pinning controllability. This allows use spectral properties of augmented Laplacian matrix, which includes the information on the control signal towards analyzing pinning controllability. Based on such information, the eigenratio of the augmented Laplacian, i.e., the largest eigenvalue divided by the smallest one, was proposed as a metric to quantify the pinning controllability of dynamical networks, i.e., the smaller the eigenratio the better the controllability^12^,^16^.

For some applications it might be desirable to have a network with high levels of pinning control. One of the important issues in optimal pinning control is to find the best driver nodes. Early works in this field selected the drivers randomly and showed that having only a small number of drivers can successfully control the whole dynamics into a reference state^12^. It was shown that controlling central nodes (e.g., those with high degree or betweenness centrality measure) is more effective than randomly selecting the drivers. Zhuo *et al.* considered the speed of synchronization to the reference trajectory, which is related to the smallest eigenvalue of the augmented Laplacian matrix^17^. Using linear matrix inequality approach, they introduced an optimization method to obtain the optimal drivers and their associated feedback gains to maximize this eigenvalue. They showed that by driving the nodes with high gains, the speed of pinning control is significantly improved as compared to the cases when nodes with high degree or betweenness centrality are chosen as drivers^17^.

We have recently proposed an optimization method to find optimal drivers and shown that it significantly outperforms heuristic approaches^16^. In this paper, we extended the previous work by further optimizing the network structure. Not only the locations of optimal driver nodes are found through an optimization technique, but also the feedback gains are optimized. We showed that optimizing the feedback gains have significant impact on the controllability. We further showed that the connection weights can also be optimized using the same optimization techniques, and the networks with optimal connection weighted have much higher controllability than unweighted ones.

## Pinning Controllability: Background Information

Controlling a complex network system has many applications ranging from engineering systems (such as power grids) to biological systems. One type of controlling a complex network is pinning control in which the dynamics of the nodes are pinned to a specific state. The ease by which a network can be controlled is often interpreted as its pinning controllability^18^,^19^. Let us consider a network with *N* nodes, in which a dynamical system seats at each node. These individual dynamical systems interact through the edges of the network and the equations of the motion read:


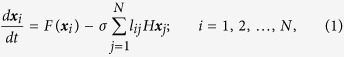


where *x*_*i *_∈ *R*^*d*^ are *d*-dimensional state vectors, *F*:*R*^*d*^ → *R*^*d*^ defines the individual dynamics of each node, *l*_*ij*_ is the element (*i*, *j*) of Laplacian matrix which describes the network topology and can be obtained from the adjacency matrix. In this work, we limit ourselves to simple (unweighted and undirected) networks, and thus entries of the adjacency matrix *A* = (*a*_*ij*_) are either 1, if there is a link between *i* and *j*, and 0 otherwise. In order to construct the Laplacian, one has to obtain *l*_*ij*_ = −*a*_*ij*_ for *i* ≠ *j*, and *l*_*ii*_ = *k*_*i*_, where *k*_*i*_ is degree of node *i. σ* is a unified coupling strength and *H* is a matrix indicating from which dimensions the dynamical systems are coupled to each other; for example, if the individual dynamical systems have three states that are connected through their first state, *H* is a 3-by-3 matrix whose (1, 1) entry is one and others are zero.

As we mentioned before, the goal of pinning control is to lead the nodes into a reference state. Let us consider a time-varying reference state as


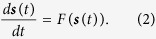


In order to pin the nodes of network to the reference trajectory *s*(*t*), often some of the nodes are chosen as drivers and the control signal is fed to them. Applying the control signal to the network, the dynamical equations read


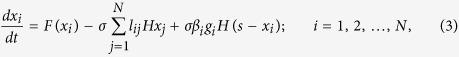


where *g*_*i*_ is the feedback control gain, and *β*_*i*_ indicates the driver nodes such that *β*_*i*_ = 1, if node *i* is a driver node and *β*_*i*_ = 0 otherwise. If certain conditions are met, the driver nodes will be able to force the network to follow the reference trajectory, and a local synchronization to this manifold is obtained provided that the initial conditions are close enough, i.e., there exists *ε* > 0 such that for the initial conditions with distance less than *ε*, one has:


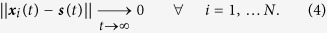


Complete synchronization to *s*(*t*) can only be attained when the individual dynamical systems are identical and some conditions are met. In general, four causes have roles in determining whether synchronization can be obtained: *i*) the dynamics of the individual systems, expressed by *F*(·) in equation (1), *ii*) the network structure, represented by the connection graph described by *A* or *L*, *iii*) the type and strength of the interaction between the individual dynamical systems, and *iv*) the driver nodes and their associated feedback control gains. In studying pinning controllability of dynamical networks, the main problem is to study the stability of the solution ***x***_1_(*t*) = ***x***_2_(*t*) = … = ***x***_*N*_(*t*) = ***s***(*t*). To this end, the master stability function mechanism, which has been originally proposed for synchronization of dynamical networks^15^, can be efficiently used. The master stability function approach gives necessary conditions for the local stability of the above synchronized solution. This approach is based on studying the variational equations; each dynamical system is considered to have small enough perturbation ***ζ***_*i*_ from the synchronous state; ***x***_*i*_(*t*) = ***s***(*t*) + ***ζ***_*i*_. The variational equations are


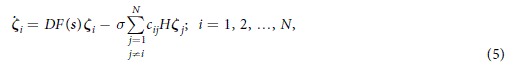


where *D* stands for Jacobian and *C* is the augmented Laplacian matrix obtained as


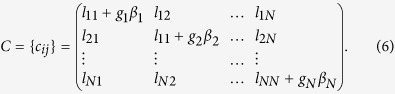


*C* has the information on the drivers (their locations and associated feedback gain) in its diagonal elements. Here, we consider undirected networks; thus *C* is a symmetric matrix (*C* = *C*^*T*^), and it can be transformed into *C* = *ΓΩΓ*^*T*^, where *Γ* is the orthogonal matrix with columns as the eigenvectors of *C* and *Ω* is a diagonal matrix of corresponding real eigenvalues. Let define ***ζ*** = (***ζ***_1_, ***ζ***_2_, …, ***ζ***_*N*_) = ***η**Γ*^*T*^, where ***η*** = (***η***_1_, ***η***_2_, …, ***η***_*N*_). Then, equation (5) is equivalent to


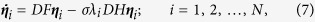


where *λ*_*i*_ are the eigenvalues of *C*, ordered as 0 < *λ*_1_ ≤ *λ*_2_ ≤ … ≤ *λ*_*N*_ 20. Since C is a symmetric matrix, all its eigenvalues take real values. A necessary condition for the stability of the synchronization manifold is that all the Lyapunov exponents of equation (7) are negative. The largest Lyapunov exponent of the variational equation (7), Λ(a) where *a* = *σλ*, called master stability function, accounts for the linear stability of the synchronization solution, i.e., if Λ(*a*) < 0, the synchronized state is stable. For many systems, the master stability function is negative only within an interval (*a*_1_, *a*_2_) where and hence, the network synchronizes to the reference trajectory in such an interval^15^. Requiring all coupling strengths to lie within this interval, i.e. *a*_1_ < *σλ*_1_ ≤ … ≤ *σλ*_*N*_ < *a*_2_, one concludes that if the network locally asymptotically synchronizes to the reference trajectory, we have


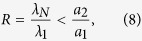


for the corresponding graph.

The left hand side of the above equation depends only the network structure and information on the drivers, whereas the right hand side depends on the individual dynamical systems and the projection matrix. *R* is accounted for pinning controllability of the network; i.e., for network *N*_1_ to be more pinning controllable than network *N*_2_, *N*_1_ should have smaller *R* as compared to *N*_2_ (provided that they have the same number of nodes and connection cost). Therefore, in order to improve the controllability one has to minimize *R*.

## Pinning Controllability: Optimization

Considering *R* as an indicator of network controllability, it depends not only on the network topology, but also on the number of driver nodes, their locations in the network and feedback gains. This way only topology of the network is important in determining its pinning control properties, and thus, controllability optimization will be performed through structural optimization of networks. In this research, we consider different scenarios to optimize *R*.

### Optimizing the locations of driver nodes (Task 1)

One of the key issues in optimizing the network structure to have high levels of pinning control over the network is the location of driver nodes. Often, there is a limited budget for control, and thus not all nodes can be driven. Indeed, it is desired to have the control done with as small number of drivers as possible. In early works, the locations of drivers were considered to be random^12^, where considering only a few drivers could successfully pin the whole network to the reference trajectory. A better way is to drive nodes with high centrality values, e.g., high-degree nodes^12^, where it has been shown that by selecting the high-degree nodes as drivers, better control performance can be achieved as compared to the case when drivers are randomly chosen. We have recently shown that one can further optimize the pinning control performance by finding the optimal locations for drivers^16^. It was shown that properties of optimal drivers significantly depend on the network type; for heterogeneous networks, optimal drivers have characteristics close to hub nodes, whereas they have properties similar to network average in homogeneous networks.

The problem of finding the best drivers can be defined as a constrained optimization problem with specific cost function. In this part, let suppose that all drivers have the same feedback gain. When the drivers are chosen based on their centrality values, first the nodes are sorted based on their centrality scores (e.g., degree or betweenness centrality), and then *N*_*d*_ 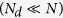 nodes with the highest centrality is considered to be drivers. Here we compare the optimization method with four heuristics including choosing the drivers based on their degree, betweenness centrality, closeness or clustering coefficient. As the feedback gains are considered to be the same, the only parameters that should be determined in the optimization process are *β*_*i*_’s that are 1 when the node is driver and 0 otherwise. Furthermore, the total number of driver nodes is *N*_*d*_. Thus, the constrained optimization problem to be solved can be formulated as


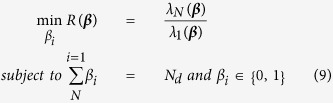


where ***β*** = (*β*_1_, *β*_2_, …, *β*_*N*_) are the parameters to be optimized. For this task, all drivers have the same feedback gain of *k*_*i*_ = 10.

### Optimizing the feedback gains (Task 2)

As mentioned, not only the locations of drivers are important in determining pinning control properties of a dynamical network, but also the feedback gains play a major role. In this section, we suppose that the locations of optimal drivers are fixed, and determine the optimal value for the feedback gains. Therefore, the only free parameters for the optimization process are *g*_*i*_’s which are considered to be in a certain interval. Thus, the optimization problem is


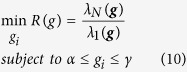


where 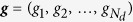 are the parameters that should be optimized. In this manuscript, the feedback gains are limited to the interval [0, 100], which means that optimal feedback gains will be in this range.

### Optimizing the locations of driver nodes and feedback gains (Task 3)

In this section we do not fix the locations of driver nodes but let the optimization process simultaneously finds the optimal locations and their associated feedback gain. Fixing the number of drivers at *N*_*d*_, *β*_*i*_’s and *g*_*i*_’s are chosen as free parameters that should be determined by the optimization process. Therefore, the optimization problem can be formulated as


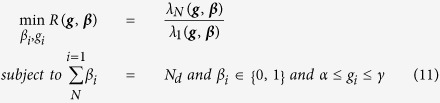


where ***β*** = (*β*_1_, *β*_2_, …, *β*_*N*_) and 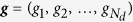 are the parameters that should be optimized. Similar to Task 2, in Task 3 also the feedback gains are limited to [0, 100].

### Optimizing link weights for better pinning control (Task 4)

In all the above problems, the underlying connection graph is an undirected and unweighted network. However, many real-world networks are weighted, and it has been shown that synchronization properties of dynamical networks can be improved by proper weighting the links^21^,^22^. Here we investigated whether the link weights can also be optimized for better control. To this end, we used the networks obtained in Task 1 (with optimized locations of drivers), and optimized the connection weights using the optimization technique. Let us denote the weight of the edge between nodes *i* and *j* by *w*_*ij*_’. The optimization problem can be formulated as


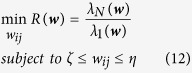


where ***w*** is a vector of edges’ weights that should be optimized. For this task, all drivers have the same feedback gain of *g*_*i*_ = 10.

### Optimization technique

The above optimization problems are not convex, and thus efficient tools available for optimization of convex problems cannot be used for them. A good approach to handle such optimization tasks is to use population-based techniques. In this paper, we used Cat Swarm Optimization (CSO), which has been shown to be an efficient population-based optimization technique^23^. CSO is an evolutionary algorithm designed to solve problems with discrete or continuous search space. This algorithm is composed of two modes: Seeking and Tracing. For modeling the behavior of cats in both the resting and alert states, we use the seeking mode. This mode is a time for thinking and deciding about the next move, and has four main parameters: seeking memory pool (SMP), seeking range of the selected dimension (SRD), counts of dimension to change (CDC) and self-position consideration (SPC). Seeking mode first makes copies of each cat (as counts of SMP by considering SPC), and then selects some copies and randomly mutates position of each cat (i.e., it selects CDC dimensions of each cat, and then mutates by (1 + SRD) or (1 − SRD) randomly)^23^. Tracing mode is responsible for moving cats to new points based on position and velocity equations considering the position of the best-performing cat. In order to integrate the two modes into the algorithm, we define a mixture ratio (MR) which indicates the rate of mixing of seeking and tracing modes. This parameter decides how many cats will be moved into the seeking mode process (the remaining cats will be moved into the tracing mode). In order to solve the first problem (i.e., finding the location of optimal driver nodes), discrete version of CSO^24^ was used, while continuous version was used to solve the second problem (i.e., finding the optimal feedback weights while the location of drivers is fixed). In order to simultaneously optimize the location of optimal drivers and their associated feedback gain, co-evolutionary technique that was introduced in^25^ was used to develop an appropriate optimization method. It is worth mentioning that other population-based optimization techniques (such as genetic algorithms, particle swarm optimization, simulated annealing and differential evolution) can also be used to solve these optimization problems; however our experiments showed that CSO resulted in better performance compared to them.

## Results and Discussion

### Datasets

The above optimization techniques are applied to both model and real networks. Pining control has potential applications in power grids in which there are generator and consumer nodes. Therefore, we used a number of benchmark and real power networks in this work including IEEE benchmark networks with various nodes, high voltage grid of Spain, France^26^ and Iran^27^ and transmission network of northern USA^28^. In these networks, a node can be a generator, transformer or substation and an edge represents a power supply line. Table 1 shows details of these networks. In IEEE benchmark networks, 14% of the nodes were considered to be drivers, while in other power grids, 5% of nodes were selected as drivers.

The experiments were also carried out on model networks. As model networks, we used preferential attachment scale-free networks, as proposed by Barabasi and Albert^29^, and small-world networks, as proposed by Watts and Strogatz^28^. Scale-free networks were constructed using the original preferential attachment algorithm introduced in^29^, which is as follows. First a small set of fully connected network is considered (here a network with 3 nodes). Then, at each step, a new node is added to the network and connected to the old nodes with a probability that is proportional to their degree, i.e., the higher the degree of a node, the higher the chance of being connected to new nodes. Each new node makes *m* connections. Networks constructed in this way will have heterogeneous degree distribution; however, not all real-world networks show such properties. In order to construct networks with homogeneous properties, we considered small-world networks and used the algorithm proposed in^28^. First, a ring graph with each node connected to its *m*-nearest neighbor is considered. Then, each edge is rewired with probability *P*. For a range of *P*, the resulting network will have short average path length, comparable to corresponding random networks, while its clustering coefficient is still high (similar to the original ring graph), that is much higher than corresponding random networks. We chose 5% of the nodes as drivers and fixed the network size as 1000 for first three tasks and 200 for the fourth one. The experiments were carried out for different values of *m* in scale-free networks and different values of rewiring probability *P* in small-world networks (with *m* = 6).

### Implementation details and parameters of the optimization method

Table 2 shows the values of the parameters of CSO method. These parameters are selected based on the recommendation from previous works^30^. The parameter “Max-Iteration” indicates the maximum number of iteration in our implementation. The optimization procedure is stopped after 500 iterations if a steady-state solution is not yet obtained. However, our experience showed that on average the optimization was terminated after 250 iterations. In order to avoid the results from random effects, we perform our experiments 20 times.

### Simulation Results

**Figure 1:**
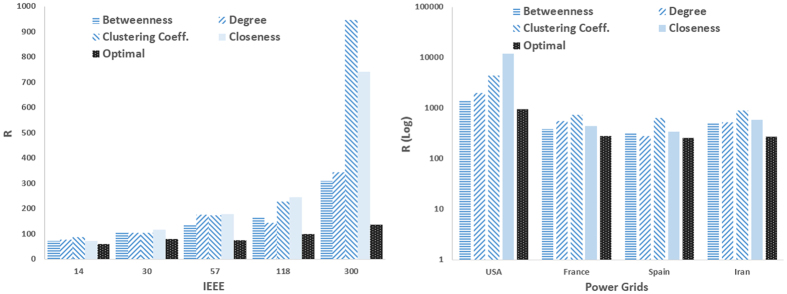
Optimal controllability (the less R the better the controllability of the network) in (left) IEEE benchmark and (right) and real power grid networks. The outcome of the optimization process (Optimal) is compared to the cases where the driver nodes are chosen based on (high) centrality values in terms of degree, betweenness, closeness, and clustering coefficient.

**Figure 2:**
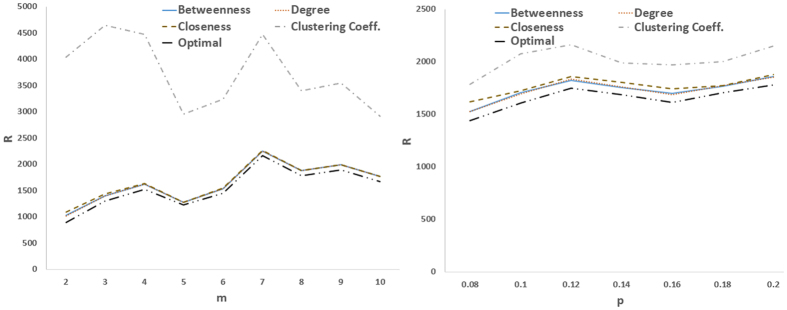
Optimal controllability as compared to heuristic methods; (left) R as a function of *m* in preferential attachment scale-free networks, and (right) R as a function of rewiring probability *P* in small-world networks. The graphs show averages over 20 realizations.

Figures 1 and 2 compare the proposed optimization method with heuristic methods (i.e., selecting the driver nodes based on their centrality scores including degree, betweenness closeness and clustering coefficient) when only the location of drivers is optimized. Figure 1 shows the result of this comparison for real networks i.e., IEEE benchmarks and real power grids. As can be seen, the optimization strategy outperforms heuristic methods by providing the best controllability, i.e., the least *R*. Among the heuristic methods, those based on closeness and clustering coefficient have the poorest performance, while degree- and betweenness-based methods are the second-top performer (after the optimal one) interchanging. The optimal one is again the best-performer in model scale-free and small-world networks (Fig. 2), while the one based on clustering coefficient has the worst performance.

**Figure 3:**
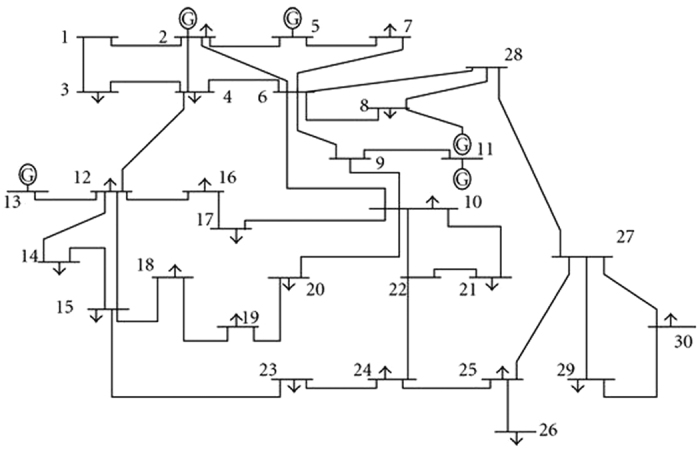
Schematic view of 30-node IEEE benchmark network.

In order to better discuss the results, we consider the 30-node IEEE benchmark network as a sample network (Fig. 3), and study properties of the optimal driver nodes. In this network, we optimize the locations and feedback gains of 4 driver nodes. In this network, the average degree of nodes is 2.7 and that of 4 hub nodes is 5.5. The proposed optimization method (Task 1) selects nodes 2, 10, 15, 27 (average degree of 4.5) as optimal drivers. Note that only nodes 2 and 10 are among the top-4 hub nodes. It is seen that sometimes taking a node with smaller degree than a hub node results in better controllability; for example, in our example node 27 with degree of 4 is better driver node than node 6 with degree 6.

**Figure 4:**
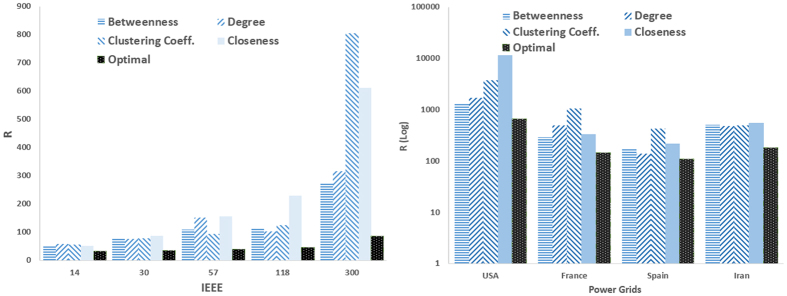
R in benchmark and real power networks when the feedback gains are optimized. The location of drivers is those of Fig. 1.

**Figure 5:**
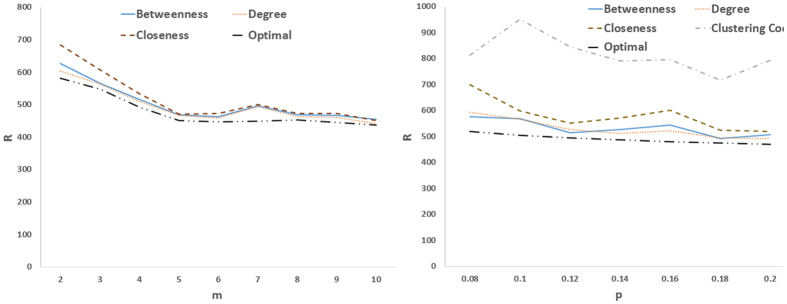
(Left) R as a function of *m* in preferential attachment scale-free networks, and (right) R as a function of rewiring probability *P* in small-world networks. The feedback gains are optimized through the optimization process and the location of drivers is those of Fig. 2. The graphs show averages over 20 realizations.

In task 2, we aimed at optimizing the feedback gains for already existing driver nodes with fixed locations, and the results are shown in Figs 4 and 5 for real and model networks, respectively. It is seen that optimizing the feedback gains have significant influence on the controllability of the networks. Comparing Figs 1 and 4 reveals that optimizing the feedback gains made almost 30% improvement in the pinning controllability of both IEEE benchmarks and real power grid networks. Considering our example network (30-node IEEE benchmark network), the optimal drivers are nodes 2, 10, 15 and 27 with optimized feedback gains as 5.93, 4.06, 6.49 and 6.38, respectively. The improvement of pinning controllability in model networks is much better; by optimizing the feedback gains, the eigenratio of scale-free and small-world networks decreased almost 60% and 70%, respectively.

**Figure 6:**
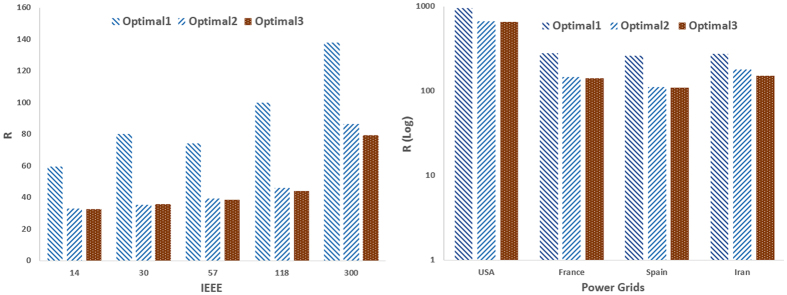
R in benchmark and real power networks when the locations and feedback gains are simultaneously optimized. The figures show the cases when only the location of drivers is optimized (Optimal 1), when only the feedback gains are optimized provided that the optimal drivers are already found (Optimal 2), and when the location of drivers and feedback gains are simultaneously optimized through a 2-dimesional optimization process (Optimal 3).

**Figure 7:**
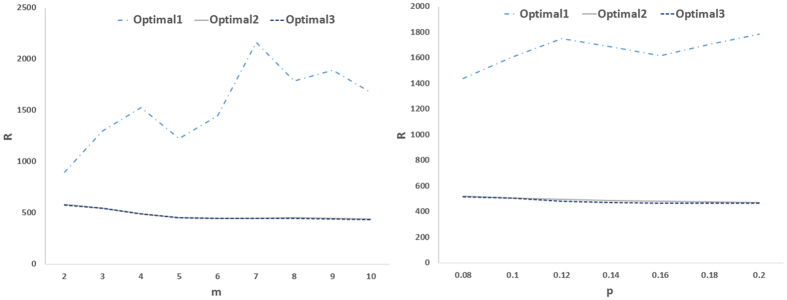
(Left) R as a function of *m* in preferential attachment scale-free networks, and (right) R as a function of rewiring probability *P* in small-world networks. Other designations are as Fig. 5 and graphs show averages over 20 realizations.

Next we consider optimizing the eigenratio as a 2-dimesional optimization problem in which both the location of drivers and feedback gains are simultaneously optimized. This allows search the solution space deeper and finds better results. Figures 6 and 7 show the results for real and model networks, respectively. It is seen that optimizing the location of drivers and feedback gains at the same time, can do the job better as compared to other cases that leads to network structures with better controllability. Another observation about the optimal controllability of model networks is that as the average degree of scale-free networks increased (i.e., *m* increased), the optimal controllability was improved. In small-world networks, as the rewiring probability increased (i.e., more shortcuts were created), communicability among the nodes improved, and as a result, the optimal controllability was enhanced.

**Figure 8:**
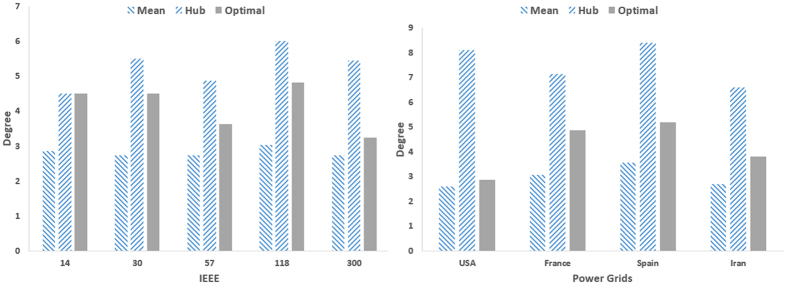
Average degree of the network (Mean), that of hub nodes (Hub), and that of optimal drivers (Optimal) in IEEE benchmark and real power networks.

**Figure 9:**
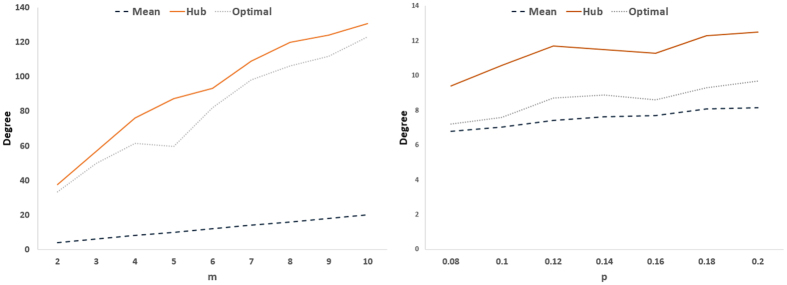
(Left) Average degree of the network (Mean), that of hub nodes (Hub), and that of optimal drivers (Optimal) in scale-free networks, and (right) average degrees as a function of *P* in small-world networks.

In order to further study details of optimal driver nodes, we compare their average degree and feedback gain with those of hub nodes as well as the whole network. For each case, only the top-*N*_*d*_ hub nodes are considered. Figures 8 and 9 compare the average degrees for real and model networks, respectively. The real networks do not have the same behavior. For example, in 14-node IEEE network, the optimal drivers are indeed the hub nodes. As the networks become larger, the average degree of optimal drivers gets closer to the average degree of the network, indicating that more hubs are missing in the set of drivers. In the largest real network (USA grid), the optimal drivers have average degree much closer to the network average than the hub nodes. Therefore, one cannot simply search for optimal drivers among the hub, and sophisticated method (like the optimization strategy proposed in this work) should be used. Model networks have also different behavior. While, the average degree of optimal drivers is close to that of hubs in scale-free networks, it is much closer to the network average in Watts-Strogatz networks. Indeed, as the network becomes more homogeneous, less hubs are selected in the optimal drivers set.

We compare the outcomes of Tasks 1, 2 and 3. Table 3 summarizes the comparisons between the outcomes of these tasks in the considered Barabasi-Albert and Watts-Strogatz networks with varying parameters. The table shows the percentage of common nodes between the outcomes of tasks 1 and 3, and also the normalized difference between the feedback gains obtained in tasks 2 and 3. As is seen, tasks 1 and 3 result in almost the same set of optimal drivers in scale-free networks for which the match is at least 90%; however it varies between 60% to 80% in small-world networks. This is mainly due to higher heterogeneity in scale-free networks. As shown in^16^, the set of optimal drivers in scale-free networks has a mean degree close to that of hub nodes (much higher than the mean degree of the whole network), while it is close to the network mean degree in small-world networks. Indeed, in scale-free networks the optimization algorithm always searches for optimal drivers within a small subset of the nodes (that are often those with high degrees), and thus varying or fixing the feedback gains has little effect on the final optimal drivers set. However, in homogeneous networks such as Watts-Strogatz small-world networks, the search space is much wider and the set of candidate nodes for optimal drivers is much larger than scale-free ones. Therefore, by optimizing the feedback gains, one will have higher degree of freedom and the set of optimal drivers may substantially change. When comparing the optimal feedback gains found in task 2 (fixed optimal drivers) and task 3 (drivers are simultaneously optimized), there is small difference (maximum 2.9%) in both scale-free and small-world networks. This indicates that optimization of feedback gain does not significantly differ whether the optimal drivers are separately fixed or simultaneously optimized with the gains.

**Figure 10:**
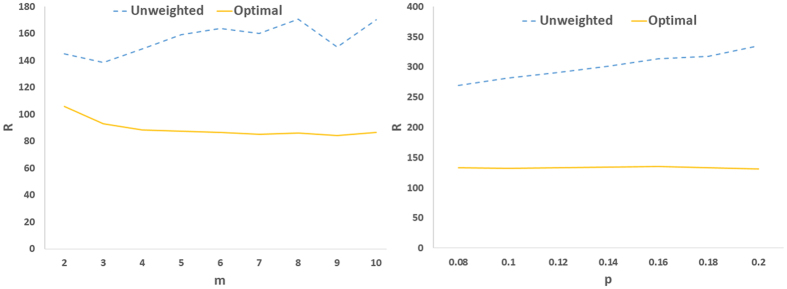
(Left) R as a function of *m* in preferential attachment scale-free networks, and (right) R as a function of rewiring probability *P* in small-world networks. The weights of existing edges are optimized through the optimization process. The graphs show averages over 20 realizations.

Task 4 is to optimize the connections weights. Figure 10 shows the influence of optimal link weighting on the controllability of model scale-free and small-world networks. Having the same feedback gain for the driver nodes, first, the locations of best drivers were found through the optimization technique, and then, the link weights were optimized. This figure compares the original unweighted network with the one with optimized weights; the optimal weighting strategy could significantly improve the controllability (more than 50% in many cases).

**Figure 11:**
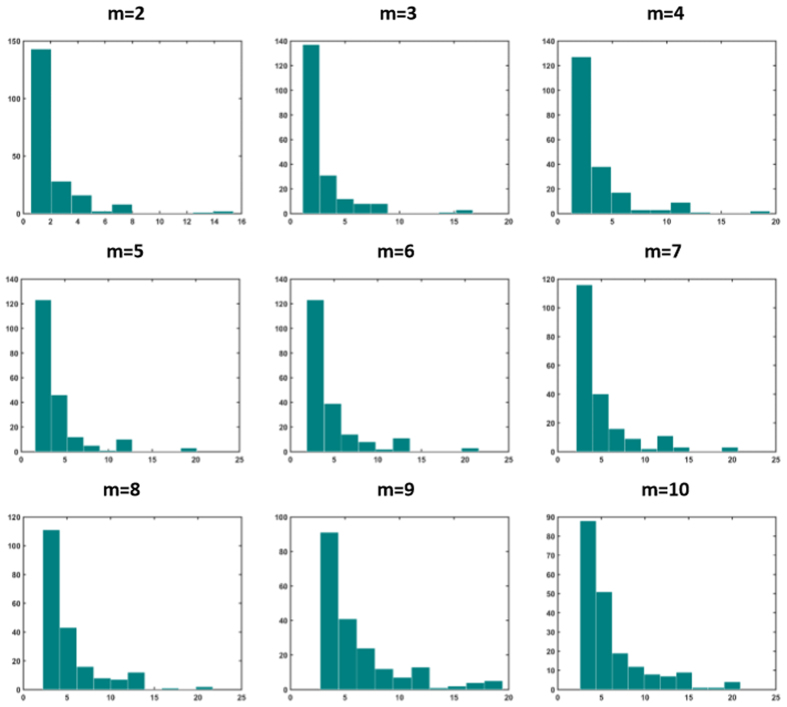
Distribution of nodal strength values in Barabasi-Albert for different values of parameter *m*. The graphs show averages over 20 realizations.

**Figure 12:**
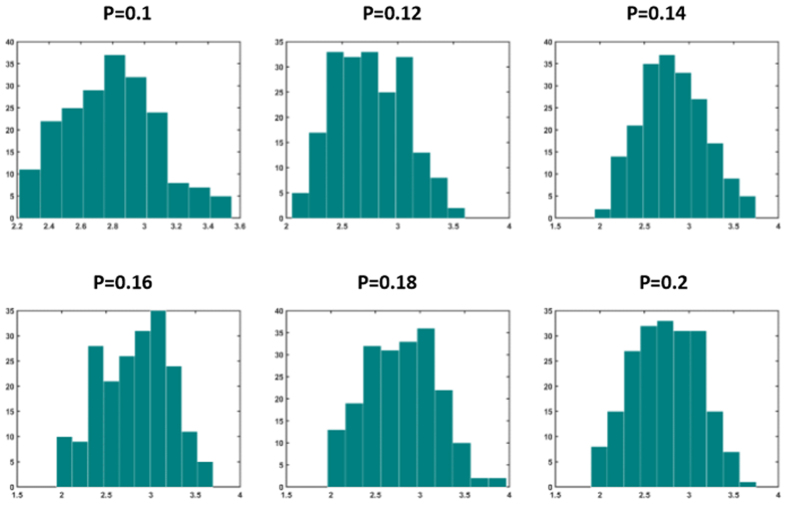
Distribution of nodal strength values in Watts-Strogatz networks for different values of rewiring probability *P*. The graphs show averages over 20 realizations.

**Figure 13:**
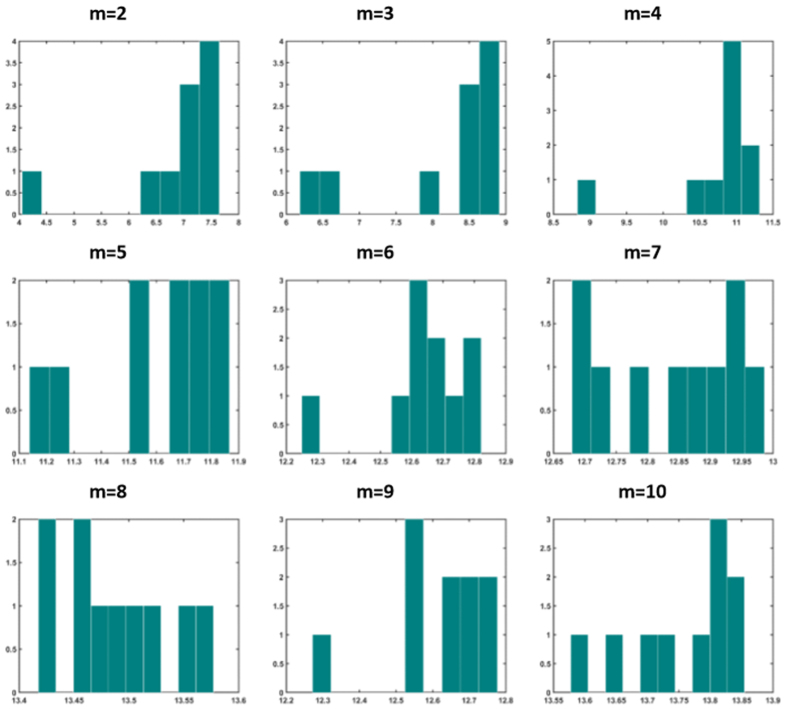
Distribution of strength values of driver nodes in Barabasi-Albert for different values of parameter *m*. The graphs show averages over 20 realizations.

**Figure 14:**
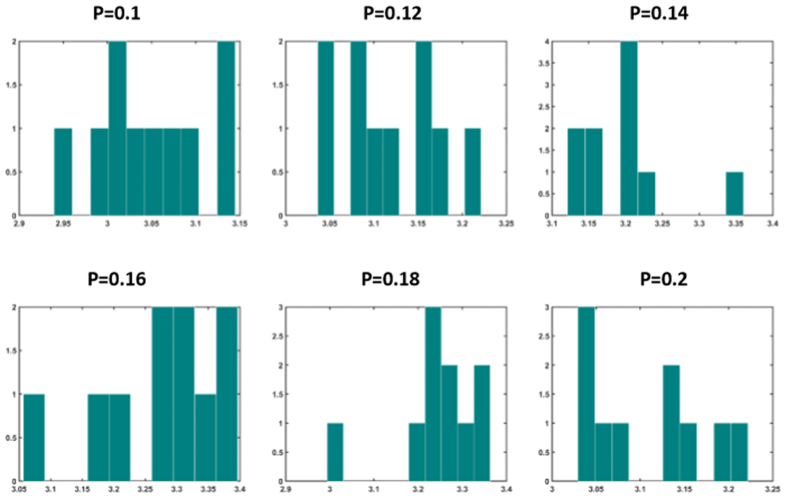
Distribution of strength values of driver nodes in Watts-Strogatz networks for different values of rewiring probability *P*. The graphs show averages over 20 realizations.

Next we study the distribution of nodes’ strength in optimally weighted networks (strength of a node is the sum of connection weights pointing to that node). It is well-known that unweighted Barabasi-Albert networks have scale-free degree distribution with exponent 3, while Watts-Strogatz networks have Poissonian degree distribution. Figures 11 and 12 show the distribution of nodal strength in optimally weighted Barabasi-Albert and Watts-Strogatz networks, respectively. These distributions are similar to the degree distribution of original networks (not shown here), but with different parameters. This indicates that the optimization strategy used in task 4 did not dramatically change the distribution of the strength values (which equals to degree in unweighted networks). However, this is not the case when the distribution is obtained for drivers (Figs 13 and 14 for Barabasi-Albert and Watts-Strogatz networks, respectively). The drivers in the optimally weighted networks have almost a flat distribution of strength values, indicating that the optimization strategy tries to make a balance between the weights connected to the drivers such that they have strength values in the same range. Indeed, the optimization process homogenizes the properties of driver nodes. In order to have deeper understanding on the distribution of optimal weights in the network, we compare the weights in hubs and leaves. To this end, the nodes are classified into two groups of hubs (high degrees) and leaves (low degrees). Table 4 shows the percentage of the hubs that have lower average link weight than the leaves. As it sees, in all cases, more than half of the hubs have lower average link weights than the leaves. This means that the optimization strategy assigns in average more weights on the links pointing to the leaves.

## Conclusions

Controlling complex network systems from an initial state to a final desired state has many applications in science and engineering. Pinning control of dynamical networks is a type of control at which the dynamics of individual nodes is pinned to a single reference trajectory. In this work, we considered a number of problems in optimal pinning controllability and used a population-based optimization technique to solve them. Using linear stability analysis and the master stability function approach, we considered a metric quantifying the controllability of the network. The metric is the largest eigenvalue of the augmented Laplacian matrix divided by its smallest eigenvalue. We then formulated four problems: *i*) optimizing the locations of driver nodes with fixed feedback gains, *ii*) optimizing the feedback gains with fixed locations for the drivers, *iii*) simultaneously optimizing the locations of drivers and their associated feedback gains, and *iv*) optimizing the connection weights. In order to solve the optimization problem, we used proper versions of cat swarm optimization technique, which is a new population-based optimization technique developed to solve non-convex optimization problems. Our extensive simulation results on both real and model networks showed that the proposed optimization method could successfully optimize the pinning controllability under different conditions. The proposed method showed superior performance as compared to a number of heuristic methods. The outcome of this research can be used where an optimal structure is required for pinning control.

## Additional Information

**How to cite this article**: Orouskhani, Y. *et al.* Optimizing Dynamical Network Structure for Pinning Control. *Sci. Rep.* **6**, 24252; doi: 10.1038/srep24252 (2016).

## Figures and Tables

**Table 1:** IEEE Benchmarks and Power Grids Details.

Network	#Nodes	#Edges	Avg. Deg.	Avg. Betweenness	Avg. Closeness	Avg. Clustering
IEEE_14_	14	20	2.85	8.92	0.0332	0.36
IEEE_30_	30	41	2.73	33.43	0.0107	0.23
IEEE_57_	57	78	2.73	110.70	0.0037	0.12
IEEE_118_	118	179	3.03	310.55	0.0014	0.16
IEEE_300_	300	408	2.73	1305.8	0.00035	0.08
France	146	223	3.05	406.58	0.0011	0.27
Spain	98	175	3.57	189.96	0.0022	0.31
Iran	105	142	2.7	305.84	0.0015	0.1
USA	4941	6594	2.6	44433	0.00001	0.08

**Table 2:** Parameter Settings for CSO method.

Parameter	Value
SMP	5
SPC	True
SRD	20%
CDC	80%
MR	2%
Population Size	250
Realization	20
Max-Iteration	500

**Table 3:** The percentage of common optimal driver nodes in the outcome of Task 1 (when only the location of drivers is optimized and Task 3 (when both location of drivers and feedback gains are simultaneously optimized), and the absolute difference (in percentage) between the feedback gains in Task 2 (when the feedback gains are optimized for the drivers found in Task 1) and Task 3.

Network	Common Nodes between tasks 1 and 3	Mean feedback gains difference between tasks 2 and 3	Network	Common Nodes between tasks 1 and 3	Mean feedback gains difference between tasks 2 and 3
BA (*m* = 2)	90%	2.3%	WS (*P* = 0.08)	60%	1.1%
BA (*m* = 3)	100%	0.6%	WS (*P* = 0.1)	80%	0.9
BA (*m* = 4)	90%	2.9%	WS (*P* = 0.12)	70%	1.4%
BA (*m* = 5)	100%	2.8%	WS (*P* = 0.14)	70%	1.6%
BA (*m* = 6)	100%	1.6%	WS (*P* = 0.16)	75%	0.7%
BA (*m* = 7)	100%	0.9%	WS (*P* = 0.18)	75%	2.4%
BA (*m* = 8)	90%	1%	WS (*P* = 0.2)	80%	1.2%
BA (*m* = 9)	90%	1.5%			
BA (*m* = 10)	90%	0.3%			

The networks are Barabasi-Albert (BA) scale-free with different m and Watts-Strogatz (WS) small-world with different rewiring probability P.

**Table 4:** The percentage of hubs that have lower average link weight than leaves.

Network	Percentage of hubs that have lower strength than leaves	Network	Percentage of link weights of hubs lower than average link weights of leaves
BA (*m* = 2)	54%	WS (*P* = 0.1)	56%
BA (*m* = 3)	51%	WS (*P* = 0.12)	62%
BA (*m* = 4)	58%	WS (*P* = 0.14)	50%
BA (*m* = 5)	59%	WS (*P* = 0.16)	55%
BA (*m* = 6)	65%	WS (*P* = 0.18)	51%
BA (*m* = 7)	55%	WS (*P* = 0.2)	51%
BA (*m* = 8)	60%		
BA (*m* = 9)	53%		
BA (*m* = 10)	58%		

The networks are Barabasi-Albert scale-free with different *m* and Watts-Strogatz small-world with different rewiring probability *P*.
